# Supplementary material for: Microbiome of Free-Living Amoebae (FLA) Isolated from Fresh Organic Produce: Potential Risk to Consumers?
Source: Foods. 2023 Aug 18;12(16):3102. doi: 10.3390/foods12163102 (PMC10453443; doi:10.3390/foods12163102)
Supplement: Supplementary file 1 [file foods-12-03102-s001.zip › foods-2538807-supplementary.pdf]

Supplementary material

Microbiome of Free-Living Amoebae (FLA) Isolated from Fresh Organic Produce: Potential Risk to Consumers?

Lara Soler [1], Yolanda Moreno [1,*], Laura Moreno-Mesonero [1,2], Inmaculada Amorós [1], José Luís Alonso [1] and María Antonia Ferrús [2]

[1] Research Institute of Water and Environmental Engineering (IIAMA), Universitat Politècnica de València, Camino de Vera s/n, 46022 Valencia, Spain; larasolergarcia@gmail.com (L.S.); laumome@upv.es (L.M.-M.); iamoros@ihdr.upv.es (I.A.); jalonso@ihdr.upv.es (J.L.A.)

[2] Biotechnology Department, Universitat Politècnica de València, Camino de Vera s/n, 46022 Valencia, Spain; mferrus@btc.upv.es

* Correspondence: ymoren@upv.es

Table S1: Relative abundance (%) of the most abundant bacterial phyla identified as part of the FLA microbiome isolated from organic fresh produce

	Cabbage	Lettuce	Spinach	Strawberry
**Proteobacteria**	71.31	57.28	39.77	82.58
**Bacteroidota**	15.97	23.03	26.90	11.24
**Verrucomicrobiota**	4.09	1.46	17.12	0.49
**Myxococcota**	3.83	9.15	4.90	0.56
**Firmicutes**	2.65	3.74	0.91	1.92
**Other**	2.15	5.34	10.40	3.22
**Total**	100.00	100.00	100.00	100.00

Table S2: Relative abundance (%) of the most abundant bacterial classes identified as part of the FLA microbiome isolated from organic fresh produce

	Cabbage	Lettuce	Spinach	Strawberry
**Gammaproteobacteria**	55.31	48.29	28.29	47.50
**Bacteroidia**	15.97	22.98	26.89	11.24
**Alphaproteobacteria**	16.00	8.99	11.49	35.07
**Verrucomicrobiae**	4.08	1.44	17.11	0.48
**Polyangia**	1.45	7.20	3.68	0.39
**Other**	7.20	11.10	12.54	5.32
**Total**	100.00	100.00	100.00	100.00

Table S3: Relative abundance (%) of the most abundant bacterial genera (>2% in at least one type of sample) identified as part of the FLA microbiome isolated from organic fresh produce

	Cabbage	Lettuce	Spinach	Strawberry
Achromobacter	5.494	1.782	1.251	0.536
Alcaligenaceae family	7.042	0.796	0.288	0.143
Alkanindiges	3.783	0.136	0.012	0.032
Allorhizobium	2.711	2.243	2.011	5.823
Aquabacterium	7.403	0.418	0.262	0.007
Brevundimonas	2.203	0.689	0.913	2.824
Caulobacter	0.286	0.609	0.113	3.071
Cellvibrio	0.475	0.914	8.407	3.397
Cupriavidus	0.159	2.120	0.015	0.022
Delftia	0.000	2.059	0.840	0.213
Devosia	0.612	0.765	2.622	0.547
Dyadobacter	2.463	2.175	2.214	0.135
Flavobacterium	1.697	10.934	12.581	1.694
Fluviicola	2.113	0.459	0.914	0.001
Massilia	2.346	5.228	0.770	1.612
Nannocystis	1.258	2.880	2.934	0.022
Novosphingobium	0.269	0.238	0.206	2.347
Pedobacter	5.416	3.674	2.911	0.399
Polyangium	0.005	4.055	0.341	0.000
Prosthecobacter	3.650	0.897	12.303	0.054
Pseudomonas	17.767	14.898	1.787	11.773
Pseudoxanthomonas	0.508	0.336	1.862	2.310
Roseomonas	2.891	0.805	1.029	1.340
Serratia	0.005	0.000	0.004	2.342
Sphingobacterium	0.529	0.279	0.362	3.327
Sphingobium	0.909	0.436	0.287	13.109
Sphingomonas	1.013	0.846	0.502	2.269
Stenotrophomonas	2.131	6.441	1.743	5.379
Variovorax	0.425	1.737	0.939	6.623
Verrucomicrobium	0.149	0.048	2.297	0.096
Xanthomonas	0.023	0.044	0.000	7.896

Table S4: Kruskal-Wallis test on all groups and pairwise comparation of the alpha diversity index Faith's Phylogenetic Diversity between types of samples.

Kruskal-Wallis (all groups)		Result		
H		15.9102		
p-value		0.0012		
**Kruskal-Wallis (pairwise)**				
**Group 1**	**Group 2**	**H**	**p-value**	**q-value**
Cabbage (n=11)	Spinach (n=11)	14.2562	0.0002	0.0010
Cabbage (n=11)	Strawberry (n=7)	0.0513	0.8209	0.8209
Cabbage (n=11)	Lettuce (n=11)	3.5023	0.0613	0.1226
Spinach (n=11)	Strawberry (n=7)	8.1388	0.0043	0.0130
Spinach (n=11)	Lettuce (n=11)	2.1829	0.1396	0.1675
Strawberry (n=7)	Lettuce (n=11)	2.2331	0.1351	0.1675

Table S5: Permanova test on all groups and pairwise comparation of the unweighted beta diversity index between types of samples.

Permanova results						
**method name**	PERMANOVA					
**test statistic name**	pseudo-F					
**sample size**	40					
**number of groups**	4					
**test statistic**	2.502					
**p-value**	0.001					
**number of permutations**	999					
**Pairwise permanova results**						
**Group 1**	**Group 2**	**Sample size**	**Permutations**	**pseudo-F**	**p-value**	**q-value**
Cabbage	Spinach	22	999	4.143	0.001	0.003
	Strawberry	18	999	2.056	0.008	0.010
	Lettuce	22	999	1.949	0.007	0.010
Spinach	Strawberry	18	999	3.829	0.001	0.003
	Lettuce	22	999	1.830	0.004	0.008
Strawberry	Lettuce	18	999	1.659	0.033	0.033

Table S6: Permanova test on all groups and pairwise comparation of the weighted beta diversity index between types of samples.

**Permanova results**	
**method name**	PERMANOVA
**test statistic name**	pseudo-F
**sample size**	40
**number of groups**	4
**test statistic**	3.558
**p-value**	0.001
**number of permutations**	999

Pairwise permanova results

**Group 1**	**Group 2**	**Sample size**	**Permutations**	**pseudo-F**	**p-value**	**q-value**
Cabbage	Spinach	22	999	4.656	0.001	0.003
	Strawberry	18	999	1.943	0.018	0.022
	Lettuce	22	999	1.818	0.053	0.053
Spinach	Strawberry	18	999	6.034	0.001	0.003
	Lettuce	22	999	3.912	0.003	0.006
Strawberry	Lettuce	18	999	2.657	0.004	0.006

Table S7: Relative abundance (%) of relevant bacterial genera identified in low abundances as part of the FLA microbiome isolated from organic fresh produce

		Acinetobacter	***Aeromonas***	***Arcobacter***	***Bacillus***	***Brevundimonas***	***Klebsiella***	***Legionella***	***Mycobacterium***	***Salmonella***
Cabbage	**C1**	2,186	0,026	0	1,909	2,450	0	0	0,010	0
	**C2**	0,674	0,018	0	7,553	0,904	0	0,118	0,347	0
	**C3**	0,055	0	0	0	1,397	0	0,005	0,034	0
	**C4**	4,879	0	0	0	1,585	0	3,560	0,016	0,039
	**C5**	0,157	0	0	0	0,533	0	0,008	0	0
	**C6**	0,577	0,024	0	0,044	0,115	0	0,885	0,128	0
	**C7**	0,128	0	0	0,086	0,313	0	0,003	0,024	0
	**C8**	0,089	0	0	0,188	1,282	0	0,010	0,044	0
	**C9**	0,029	0	0,008	0,703	3,492	0	0	0,481	0
	**C10**	0,021	0	0	0,159	6,893	0	0,016	0,133	0,206
	**C11**	0,293	0	0,013	7,572	5,265	0	0	0,068	0
Lettuce	**L1**	0,167	0	0	0,086	0,209	0	6,180	0,206	0
	**L2**	2,413	0,274	0	0,770	0,509	0,253	5,208	0	0
	**L3A**	0,964	0	0	0	1,382	0	0	0,029	0,047
	**L4**	3,233	0	0	0	1,745	0	0	0	0
	**L5**	3,069	0	0	3,213	0,005	0	0	0	0
	**L6**	0,050	0	0	0,068	0,146	0	0	0,008	0
	**L7**	0,697	0	0	1,499	0,039	0	0,029	0,008	0
	**L8**	0,052	0,010	0	0,018	0,178	0	0,794	0,084	0
	**L9**	0,021	0	0,005	0	0,248	0	0	0	0
	**L10**	0,308	0	0	0	2,834	0	0,008	0	0
	**L11**	0,690	0,138	0	0	0,279	0	0,076	0,005	0
Spinach	**Sp1**	0	0	0	0	1,836	0	0,008	0	0
	**Sp2**	2,014	0,966	0	0	1,403	0	0,345	0,055	0,005
	**Sp3**	0	0,010	0	0	0,212	0,039	0,115	0,078	0
	**Sp4**	0,052	0,081	0	0,363	0,050	0	0,248	0,047	0
	**Sp5**	0,481	0,039	0	0	0,799	0	0,196	0	0
	**Sp6**	0,016	0,039	0	0	0,214	0	0,052	0,008	0
	**Sp7**	0,039	0,256	0	0,044	0,559	0	0,044	0,008	0
	**Sp8**	0,037	0	0	0	2,110	0	0	0	0
	**Sp9**	0,951	0,548	0	0	0,765	0	0	0	0
	**Sp10**	0,044	0,123	0	0	0,340	0	0	0	0
	**Sp11**	0,136	0,037	0	0	1,760	0	0,047	0,010	0
Strawberry	**St1**	0	0	0	0,089	0,052	0	0	0	0
	**St2**	0,332	0	0	0	3,048	0	0	0	0
	**St3**	0,065	0	0	0	2,844	0	0,005	0	0,018
	**St4**	0	0	0	0	5,696	0	0	0	0
	**St5**	0,003	0	0	0,324	0,266	0	0,003	0,010	0
	**St6**	0,008	0,013	0	0,055	6,895	0	0,123	0	0
	**St7**	0	0	0	0	0,966	0	0,836	0	0
